# Bioinspired photonic structures by the reflector layer of firefly lantern for highly efficient chemiluminescence

**DOI:** 10.1038/srep12965

**Published:** 2015-08-12

**Authors:** Linfeng Chen, Xiaodi Shi, Mingzhu Li, Junping Hu, Shufeng Sun, Bin Su, Yongqiang Wen, Dong Han, Lei Jiang, Yanlin Song

**Affiliations:** 1 Key Laboratory of Green Printing, Key Laboratory of Organic Solid, Beijing National Laboratory for Molecular Sciences (BNLMS), Institute of Chemistry, Chinese Academy of Sciences, Beijing 100190, P. R. China; 2 China Research Institute for Science Popularization (CRISP), Beijing 100081, P. R. China; 3 Research Center for Bioengineering & Sensing Technology, University of Science and Technology Beijing, Beijing 100083, P. R. China; 4 National Center for Nanoscience and Technology, Beijing 100190, P. R. China; 5 Institute of Biophysics, Chinese Academy of Sciences, Beijing 100101, P. R. China

## Abstract

Fireflies have drawn considerable attention for thousands of years due to their highly efficient bioluminescence, which is important for fundamental research and photonic applications. However, there are few reports on the reflector layer (RL) of firefly lantern, which contributes to the bright luminescence. Here we presented the detailed microstructure of the RL consisting of random hollow granules, which had high reflectance in the range from 450 nm to 800 nm. Inspired by the firefly lantern, artificial films with high reflectance in the visible region were fabricated using hollow silica microparticles mimicking the structure of the RL. Additionally, the bioinspired structures provided an efficient RL for the chemiluminescence system and could substantially enhance the initial chemiluminescence intensity. The work not only provides new insight into the bright bioluminescence of fireflies, but also is importance for the design of photonic materials for theranostics, detection, and imaging.

Organisms with unique photonic functions, such as iridescent colors^1^,^2^,^3^,^4^, antireflection^5^,^6^, and optical waveguide^7^,^8^, provide inspirations for researchers to design various functional photonic materials, which hold great potential in lasers^9^,^10^, detection^11^,^12^, optical fibers^13^,^14^,^15^,^16^, and displaying^17^,^18^. For instance, the iridescent colors arising from periodic structures, e.g. peacock feathers^2^, Polia fruits^3^, and opals^19^, inspired photonic crystals, boosting the development of detection, displaying and lasers. The brittlestars with characteristic calcite double-lens in arms are sensitive to light^20^, providing new ideas for designing novel materials with both mechanical and optical functions.

As the typical species of bioluminescence, fireflies (Coleoptera: Lampyridae) have attracted human attention for their “lanterns” (light-generating organs, LOs) for thousands of years^21^,^22^,^23^,^24^,^25^. In order to fulfill their life missions, such as warning, mating and predation^26^,^27^, fireflies communicate with each other by delivering bright bioluminescence signals from LOs in the dark night. The unique structure of LOs featured to be composed of a cuticle, a photogenic layer (PL), and a reflector layer (RL) is dedicated to the bright luminescence^28^. As reported, the photochemical reaction in the PL is highly efficient (quantum yield ~41%)^29^. Besides the internal quantum efficiency, the light out-coupling efficiency is another crucial factor for the lighting efficiency. Recently, Kim *et al.* reported that the ordered nanostructures on the cuticle could help to efficiently extract the bioluminescence light^30^. But until now, there is a lack of reports on the RL.

Herein, we showed the unique structure of the RL, which was packed with hollow microgranules in an average diameter of 1.12 μm and had high reflectance in the visible range. Inspiredly, the artificial films with high reflectance were fabricated using hollow silica microparticles, which could efficiently enhance the initial chemiluminescence intensity. When the thickness of the photonic structure was about 46 μm, the chemiluminescence intensity was increased up to 55.3 times.

## Results

### The detailed microstructure of the reflector layer

**Figure 1:**
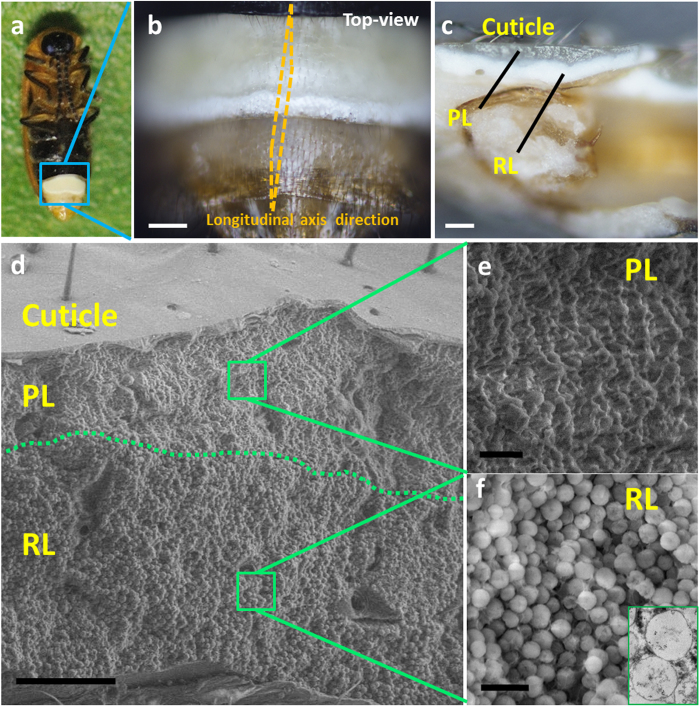
The appearance and detailed structure of the firefly lantern. (**a**) Abdominal view of a firefly. Photograph courtesy of L.F.C. (**b**) The optical image of the white light organ. The scale bar is 100 μm. (**c**) Side-view of longitudinal-section of the light organ composed of a cuticle (the top), a photogenic layer (PL, the dark part), and a reflector layer (RL, the white part). The scale bar is 50 μm. (**d**) The SEM image of the firefly lantern, confirming the three-layered structure. The scale bar is 20 μm. (**e**) SEM magnification of the PL, showing the irregular structure. The scale bar is 2 μm. (**f**) SEM of the RL, presenting the granule structure. The inset was the TEM image of an ultramicrotomed sample with section of ca. 70 nm, indicating the hollow structure. The scale bar is 2 μm.

The fireflies were collected in Beijing (China) in summer. With the whole bodies in the length of ca. 5 mm, their backs of fireflies are khaki (Fig. S1) and the ventral surfaces are black except for the white LOs (Fig. 1a,b). In order to see the inner structure, the LOs were cut along the longitudinal axis (Fig. 1b) after dehydration, which were then observed by an optical microscope. As shown in Fig. 1c, the LOs consisted of three layers, namely, a cuticle (the top), a PL (the dark part), and a RL (the white part). The three-layered structure of LOs was further confirmed by scanning electron microscopy (SEM), which indicated the thickness of the RL was ca. 40 μm, as shown in Fig. 1d. The SEM magnification images gave the detailed structure of the PL and the RL (Fig. 1e,f). The RL was composed of round granules with an average diameter of 1.12 μm, while the PL was irregular structures. Remarkably, the micro granules in the RL were found to be hollow by transmission electron microscopy (TEM) (Fig. 1f, the inset), which was rarely discovered in nature. The hollow structure was also confirmed by SEM and atomic force microscopy (AFM) (Fig. S2).

**Figure 2:**
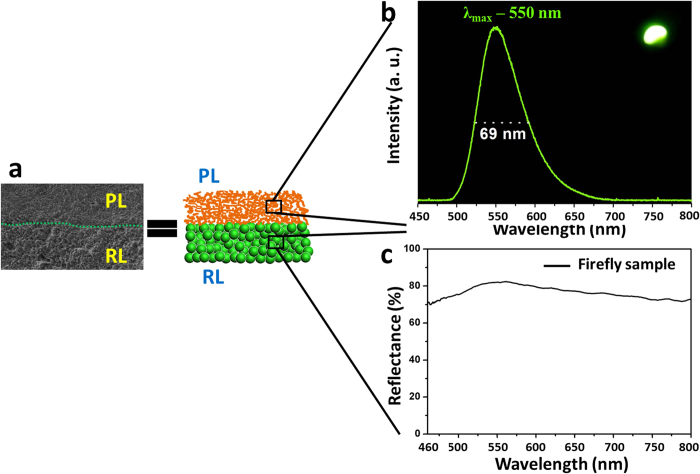
The spectra of firefly bioluminescence and reflectance of the reflector layer. (**a**) A simplified model of the firefly lantern based on the SEM observation. (**b**) Fireflies emit bright bioluminescence in the dark (the inset) with the central wavelength locating at ca. 550 nm. (**c**) The reflector layer of fireflies exhibited high reflectance in the visible region.

Fireflies emitted bright luminescence at night with the central wavelength locating at about 550 nm (Fig. 2b), and the full width at half maximum was ca. 69 nm. The bioluminescence is highly efficient, which is usually ascribed to the catalytic reactions in the PL^29^. Although the RL was also proposed to contribute to the bioluminescence by reflecting the light emitted from the PL^28^,^31^, its detailed structure and function have not been confirmed. We found that the RL exhibited high reflectance in the range from 400 nm to 800 nm (Fig. 2c). Especially, the reflectance reached up to 82% at ca. 550 nm. The unique structure found in the RL was believed to be crucial for the bright bioluminescence. Based on the structure of the LOs, a simplified model was proposed (Fig. 2a). The highly reflective property indicated the RL could reflect biofluorescence from the PL and enhance the light intensity. As the band width of bioluminescence was large, the RL acted as a reflective platform, which reflected light in a wide range of wavelength. The RL provided the inspiration to design new photonic structures which could efficiently enhance the chemiluminescence intensity, as shown below.

### Artificial photonic structure construction and its reflective property

**Figure 3:**
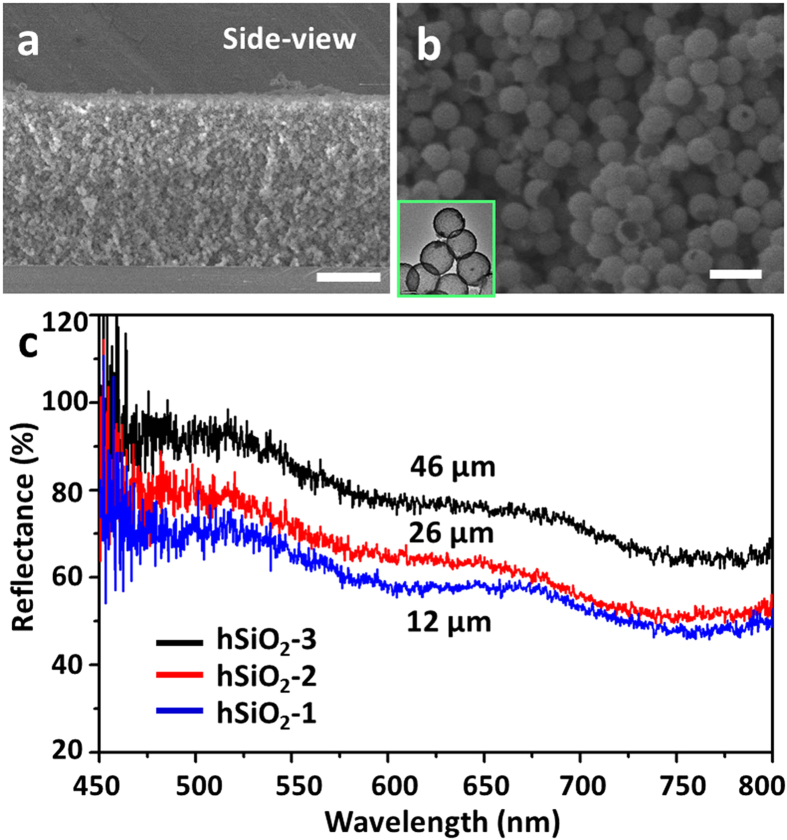
The structure and reflective property of artificial films. (**a**) Side-view of the artificial structure composed of ca. 1.05 μm hollow silica by SEM. The scale bar is 20 μm. (**b**) SEM magnification of (**a**) showing the hollow particle structure. The inset was the TEM image of hollow silica particles. The scale bar is 2 μm. (**c**) The artificial film hSiO_2_-1 (ca. 12 μm), hSiO_2_-2 (ca. 26 μm), and hSiO_2_-3 (ca. 46 μm) exhibited high reflectance in the visible region.

The preparation of hollow silica particles was achieved by a modified template method according to the literature^32^, and the process was described in Fig. S3a. Polystyrene (PS) microparticles were first prepared, which were subsequently coated by silica (PS@SiO_2_). After removal of PS cores by extraction, hollow silica (hSiO_2_) microparticles were obtained. Fig. S3b exhibited the SEM images of as-prepared PS (ca. 1.10 μm), PS@SiO_2_ (ca. 1.06 μm), and hSiO_2_ (ca. 1.05 μm). Then, hSiO_2_ particle latex was deposited on a cover glass to get the artificial reflection film mimicking the structure of RL in LOs. By controlling the concentration of the particle solution, a series of reflection films with different thicknesses would be achieved. Figure 3 presented the structure of an artificial film, and the hollow structure of hSiO_2_ was clearly observed by SEM and TEM.

Similar to the RL of LOs, the artificial reflection films exhibited high reflectance in the visible region. We fabricated the films of hSiO_2_-1, hSiO_2_-2, and hSiO_2_-3 with the thickness of ca. 12 μm, ca. 26 μm, and ca. 46 μm, respectively. As demonstrated in Fig. 3c, the reflectance of the artificial structures was dependent upon the thickness of the structures, and reached up to nearly 93% when the thickness was ca. 46 μm (black curve).

### Photonic structures enhanced the chemiluminescence

**Figure 4:**
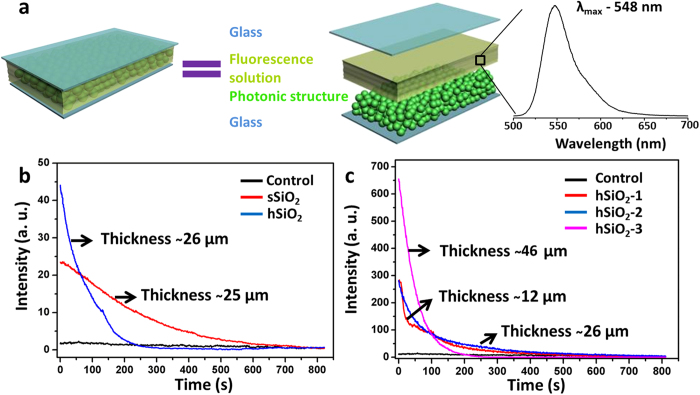
The effect of bioinspired photonic structures on chemiluminescence systems. (**a**) The scheme illustrating the studying device composed of a bottom cover glass, random hollow silica particles, the chemiluminescence solution, and a top cover glass. The chemiluminescence system could emit light with the central wavelength at about 548 nm; (**b**) The chemiluminescence intensity versus time by photonic structures consisting of solid silica particles (ca. 1.08 μm in diameter and 25 μm in thickness, red curve) and hollow silica particles (ca. 1.05 μm in diameter and 26 μm in thickness, blue curve); (**c**) The chemiluminescence intensity versus time by the control and photonic structures with the thickness of 12 µm (hSiO2-1), 26 µm (hSiO2-2), and 46 µm (hSiO2-3) on average.

In order to investigate the property of such structures consisting of hSiO_2_, the Rubrene-bis(2-carbopentyloxy-3, 5, 6-trichlorophenyl)oxalate (CPPO)-H_2_O_2_ chemiluminescence system was selected (Fig. S4)^33^. In the presence of H_2_O_2_, CPPO would be oxidized to the four-ring intermediate. Subsequently, the four-ring intermediate transferred the energy to rubrene (an excited-state) which would release the energy as light^34^. The central wavelength of the chemiluminescence located at about 548 nm, which was similar to the bioluminescence of fireflies. The prepared chemiluminescence reaction cell was a sandwich structure with an artificial RL in the middle layer, as shown in Fig. 4a. The hSiO_2_ particles with an average diameter of 1.05 μm were precipitated on the bottom cover glass, and the top cover glass was used to cover the structure. The Rubrene-CPPO-H_2_O_2_ system was then injected into the space between the two cover glasses, and the intensity of chemiluminescence versus time was detected with fluorescence spectrometer. As a control experiment, the sample without the hSiO_2_ structure was also performed. As demonstrated in Fig. 4b, the initial intensity of chemiluminescence of the sample with hSiO_2_ (ca. 26 μm in thickness) was greatly enhanced as high as 26.3 times compared to that without hSiO_2_. The results indicated the structure with hSiO_2_ played a special role in the chemiluminescence system. As another control, the structure composed of solid silica particles (sSiO_2_) with diameter 1.08 μm on average (ca. 25 μm in thickness) was also studied. As illustrated in Fig. 4b, the initial chemiluminescence intensity could also be enhanced by the sSiO_2_ film by a factor of ca. 14.0, which was less than that composed of hSiO_2_. The results showed that the unique structure mimicking the RL of LOs presented superior performance to enhance the intensity of emitted light. The bioinspired structure displayed high reflectance in a broad wavelength region, which acted as a reflector to boost the chemiluminescence light extraction. In addition, the higher surface area to volume ratio of hollow particle enhanced the mass transfer in the interface^33^,^35^, which accelerated the reaction rate, resulting in the increase of chemiluminescence intensity.

In addition, the optical effect of the chemiluminescecne reaction cell was also related with the as-prepared photonic structure thickness. As exhibited in Fig. 4c, the films of 1.05 μm hSiO_2_ in the thickness of ca. 12 μm (hSiO_2_-1), 26 μm (hSiO_2_-2), and 46 μm (hSiO_2_-3) were investigated. All the samples displayed a significant increase of the initial chemiluminescence intensity than the control sample. Furthermore, the chemiluminescence could be tuned by the thickness of the structure. With the increase of thickness, the initial chemiluminescence intensity was increased accordingly. The emission intensity could be enhanced up to 55.3 times when the thickness was ca. 46 μm. The bioinspired investigation will provide new insight into understanding the function of the RL of LOs on bioluminescence, and the design of photonic materials for chemiluminescence systems, which is promising for the development of detection, imaging, light sources, and reflectors.

## Methods

### Materials

Glutaraldehyde (25%), rubrene (97%), bis(2-carbopentyloxy-3, 5, 6-trichlorophenyl) oxalate (CPPO, 95%), 2-methacryloyloxy ethyltrimethylammonium chloride (MTC, 72%), Poly(vinyl pyrrolidone) (PVP, K30), are purchased from Alfa Aesar. 2,2-Azoisobutyronitrile (AIBN, 99%) was purchased from J&K company. H_2_O_2_ (30 w%), styrene (99%), tetraethoxysilane (TEOS, 28%), ammonium (25 w%), and ethanol (99%) were purchased from Beijing chemical works. Styrene was used after distillation. All the other chemicals were directly used without further treatment.

### Instruments and characterizations

The light images were obtained with a light microscope (Olympus BX 51) attached with a color digital CCD camera (Nikon DS-Ri1 CCD). The fluorescence images were taken by a camera. Scanning electron microscopy (SEM) was performed using Quanta 200 FEG at 3 kV under low vacuum conditions. Atomic force microscopy (AFM) was taken on SPA 400 with the tapping mode. Transmission electron microscopy (TEM) was carried out on Philips CM operated at 200 kV. The bioluminescence of fireflies and the reflectance of the reflector layer were recorded by Ocean HR 4000 fiber optic UV-Vis spectrometer. The fluorescence spectra were collected by UV-4100 spectrometer.

### Treatment of the lanterns of fireflies

Fireflies (Coleoptera: Lampyridae) for this study were collected between June and September in Haidian district of Beijing, China. The lanterns were directly cut off and immersed in pH 7.2 phosphate buffer solution containing 2.5% glutaraldehyde (the fixing fluids), and stored in the fridge at 4 °C before further treatments. After fixation, the lanterns were washed, dehydrated with ethanol solution (50%, 15 min; 75%, 15 min; 85%, 15 min; 95%, 15 min; 100%, 15 min), and finally dried by the method of CO_2_ critical point drying. The samples for SEM characterization and reflective detection were obtained by treatment of the lantern in liquid nitrogen. For TEM detection, the fixed lantern was washed with phosphate buffer solution for 3 times, followed by dehydration with ethanol. Then the lantern was embedded in EPON epoxy resin, which was treated at 60 °C for 24 h. Finally, the lantern embedded in epoxy resin was cut by ultramicrotome (Leica EM UC6) with ca. 70 nm in thickness, and collected on TEM grid.

### Preparation of hollow silica particles

Hollow silica (hSiO_2_) particles were prepared by a template method^32^. Taking the preparation of hSiO_2_ with an average diameter of 1.05 μm as an example, polystyrene (PS) latex particles were firstly prepared by the emulsion polymerization method. Styrene (4.1 mL), ethanol (28 mL), H_2_O (5 mL), and stabilizer PVP (1.0 g) were charged into a three-necked flask. The mixture was stirred by a mechanical stirrer and deoxygenated for about half an hour at 20 °C. Then the temperature increased to 70 °C, and AIBN (0.152 g) was added. After reaction for 1.5 h, the mixture of styrene (4.1 mL), ethanol (27 mL), and MTC (355 μL) was added into the flask. After 2 h, ammonium (2 mL) was added and the temperature was decreased to 50 °C, followed by the addition of TEOS (1.5 mL), which was reacted for 1.5 h. Finally, PS coated with silica (PS@SiO_2_) was obtained by centrifugation. The further treatment of PS@SiO_2_ by toluene would give the hSiO_2_. By changing the amount of PVP and AIBN, hSiO_2_ with different diameters would be achieved.

### Preparation of artificial structures

The confocal microscopy culture plates with cover glasses (completely washed) attached as the bottom were used as the devices to prepare artificial photonic structures. The center of the plate was filled with the hollow silica solution (pH ~ 7, 350 μL). After the water was evaporated in room temperature, artificial structures mounting on the cover glasses were obtained. The thicknesses of photonic structures could be controlled by the concentration of particle solutions. The control sample composed of solid silica particles was fabricated by the similar procedure.

### Detection of the chemiluminescence

The experiments were carried out at ca. 18 °C. The obtained artificial structure was covered by the other cover glass (completely washed). Subsequently, the tertiary butanol solution (20 μL) containing H_2_O_2_ (2 v%), CPPO (5 × 10^−3^ M), and rubrene (5 × 10^−5^ M) was added into the space between two cover glasses. The chemiluminescene versus time was recorded by UV-4100 spectrometer.

## Additional Information

**How to cite this article**: Chen, L. *et al.* Bioinspired photonic structures by the reflector layer of firefly lantern for highly efficient chemiluminescence. *Sci. Rep.* **5**, 12965; doi: 10.1038/srep12965 (2015).

## Supplementary Material

Supplementary Information
