# Supplementary material for: Bioinspired photonic structures by the reflector layer of firefly lantern for highly efficient chemiluminescence
Source: Sci Rep. 2015 Aug 12;5:12965. doi: 10.1038/srep12965 (PMC4532992; doi:10.1038/srep12965)
Supplement: Supplementary Information [file srep12965-s1.pdf]

Supporting information

Bioinspired photonic structures by the reflector layer of firefly lantern for highly efficient luminescence

Linfeng Chen,[1,&] Xiaodi Shi,[1,&] Mingzhu Li,[1,*] Junping Hu,[2] Shufeng Sun,[5] Bin Su,[1] Yongqiang Wen,[3] Dong Han,[4] Lei Jiang,[1] & Yanlin Song,[1,*]

[1]Key Laboratory of Green Printing, Key Laboratory of Organic Solid, Beijing National Laboratory for Molecular Sciences (BNLMS), Institute of Chemistry, Chinese Academy of Sciences, Beijing 100190, P. R. China

[2]China Research Institute for Science Popularization (CRISP), Beijing 100081, P. R. China

[3]Research Center for Bioengineering & Sensing Technology, University of Science and Technology Beijing, Beijing 100083, P. R. China

[4]National Center for Nanoscience and Technology, Beijing 100190, P. R. China

[5]Institute of Biophysics, Chinese Academy of Sciences, Beijing 100101, P. R. China

Email: ylsong@iccas.ac.cn, mingzhu@iccas.ac.cn

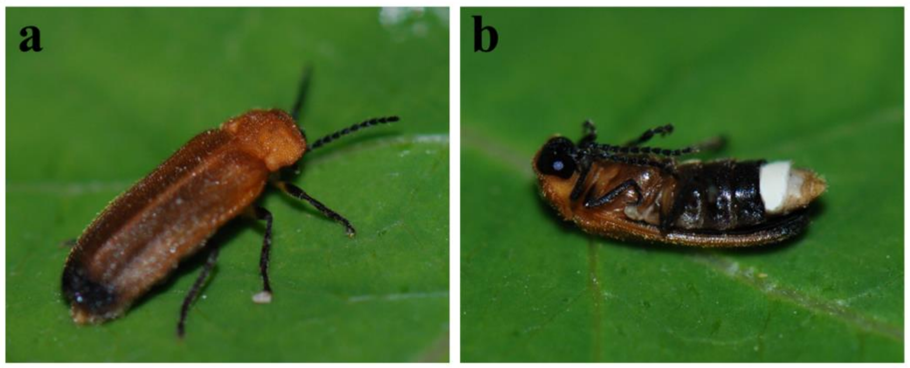

Figure S1. Photograph of a firefly. The back of the firefly is khaki, and the ventral surface is black except for the white light organ. (Photographs courtesy of L.F.C.)

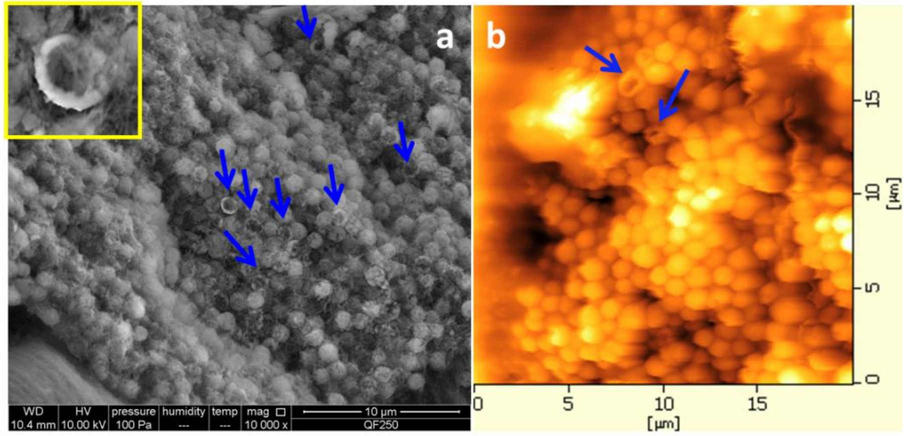

Figure S2. The SEM (a) and AFM (b) of the reflector layer of firefly lantern showing the hollow structure of granules

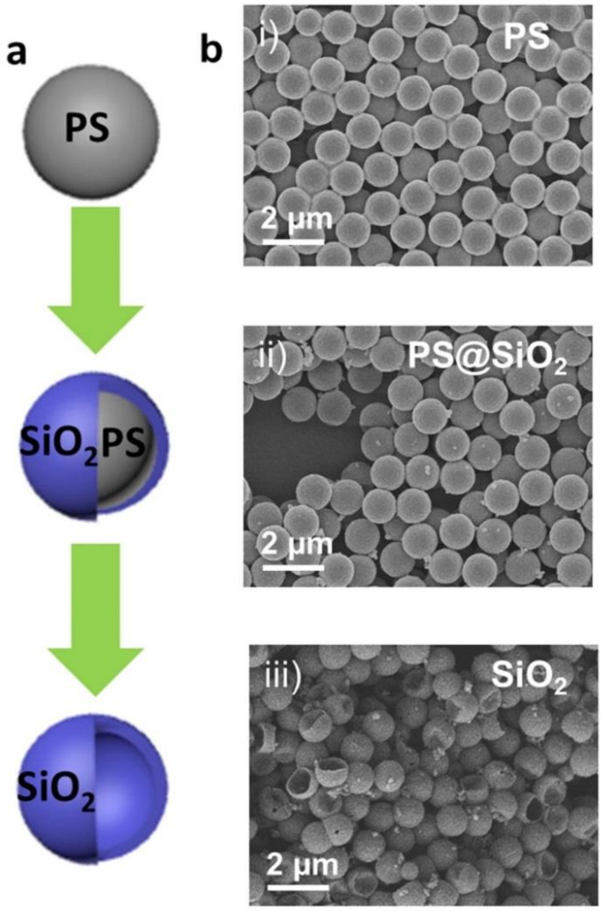

Figure S3. (a) The scheme illustrating the process of preparation of hollow silica particles; (b) SEM of PS, PS@SiO_2, and hSiO_2

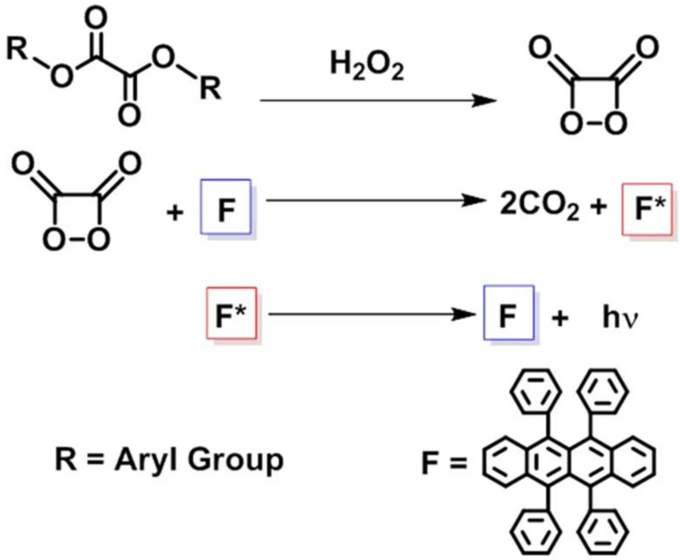

Figure S4. The system of chemiluminescence. Bis(2-carbopentyloxy-3, 5, 6-trichlorophenyl)oxalate (CPPO) was oxidized by H_2O_2, and the activated fluorescence rubrene which emitted light when transformation from the excited state to the ground state.
